# Bayesian hierarchical modelling of academic orientation and advising effects on student retention and progression: Multi-cohort evidence

**DOI:** 10.1371/journal.pone.0345001

**Published:** 2026-03-17

**Authors:** Moeketsi Mosia, Lerato Sekonyela, Felix O. Egara, Eli Nimy, Irvin M. Mabokgole, Fadip A. Nannim

**Affiliations:** 1 Department of Mathematics, Natural Sciences and Technology Education, University of the Free State, Bloemfontein, South Africa; 2 Centre for Teaching, Learning and Programme Development, Sol Plaatje University, Kimberley, South Africa; 3 Department of Mathematical Sciences, Sol Plaatje University, Kimberley, South Africa; Northwestern Polytechnical University School of Software and Microelectronics, CHINA

## Abstract

Student retention and academic progression remain central concerns in higher education, particularly within contexts characterised by widening access and structural inequality. This study examines the independent and interactive effects of academic orientation performance and academic advising utilisation on first-year student retention and progression at a South African public university. Using administrative data from 8,300 undergraduate students across three entry cohorts spanning the 2023–2024 academic periods, we employ Bayesian hierarchical multivariate modelling to account for cohort-level variation and the interdependence between retention and progression outcomes. Retention is operationalised as enrolment in the subsequent academic year and is analysed only for cohorts with observable follow-up data (based on confirmed registration records), while academic progression is examined for all cohorts. Results indicate that stronger performance in academic orientation is positively associated with both retention and progression, while engagement with academic advising is associated with improved outcomes, particularly when combined with higher orientation performance. The observed interaction effects suggest complementary engagement between orientation and advising rather than differential treatment effectiveness, and all estimated relationships are interpreted as associative rather than causal. Model comparison results indicate statistically indistinguishable predictive performance between joint and univariate specifications, with the joint model offering additional inferential advantages by capturing correlations between outcomes. Overall, the findings highlight the value of integrated first-year support interventions and demonstrate the utility of Bayesian hierarchical approaches for analysing complex, multi-cohort educational data in Global South higher education contexts.

## Introduction

Student retention and academic progression remain critical areas of concern in higher education, particularly during the transition from secondary to tertiary study [1,2]. Universities worldwide are under increasing pressure to improve student outcomes through evidence-based support systems, especially for first-year undergraduates who are most at risk of attrition [3]. Among the most widely implemented interventions are academic orientation programmes and academic advising services [4–6]. Orientation programmes are designed to assist students in acclimatising to the academic and social demands of university life by introducing them to institutional expectations, academic skills, and available support resources [7]. Academic advising, in contrast, offers personalised guidance, planning, and mentoring throughout the student journey and has been linked to greater academic success and institutional commitment [8].

While these interventions have been studied individually, there remains a lack of robust empirical evidence examining their combined effects on student retention and progression, particularly across multiple cohorts. Moreover, most existing research adopts cross-sectional or single-cohort approaches, limiting the generalisability of findings and failing to account for contextual and temporal variations in intervention effectiveness. As a result, higher education leaders lack reliable, longitudinal insights to inform student support policies. This study addresses these limitations by applying Bayesian hierarchical modelling to institutional administrative data from 8,300 first-year undergraduate students enrolled in a mandatory academic orientation course between 2023 and 2025 at a South African university. The modelling framework enables the estimation of individual-level effects (e.g., orientation performance and advising utilisation) and cohort-level variation, while accounting for potential interaction effects between the two interventions. In doing so, the study aims to inform more adaptive, data-driven student success strategies that reflect institutional and cohort-specific dynamics. The following are the research questions (RQs) and hypotheses (H₀) formulated to guide the study.

### Research questions

1. What is the probabilistic relationship between academic orientation performance, academic advising utilisation, and student retention outcomes?
2. How do academic orientation performance and academic advising utilisation influence student academic progression?
3. Do the relationships between academic orientation, advising interventions, and student outcomes vary systematically across student cohorts?
4. Which Bayesian modelling approach (univariate vs. multivariate) provides superior predictive performance for student success outcomes?

### Hypotheses

1. Higher academic orientation performance is positively associated with student retention.
2. Academic advising utilisation increases the likelihood of student retention.
3. Higher academic orientation performance is positively associated with academic progression.
4. Academic advising utilisation increases the likelihood of academic progression.
5. There is a statistically significant interaction effect between orientation performance and advising on retention and progression.
6. The effects of orientation and advising vary across student cohorts.
7. A joint multivariate Bayesian model will outperform or offer additional insights compared to separate univariate models.

### Theoretical framework

This study is grounded in established theoretical perspectives that collectively offer a coherent basis for understanding how structured academic support interventions influence student retention and academic progression. Central to this framework is Tinto’s Theory of Student Departure, originally formulated in 1993 and further elaborated in his later work [3]. Tinto posits that students are more likely to persist in higher education when effectively integrated into university life’s academic and social dimensions. Recent empirical evidence reinforces this theoretical proposition. Rehman et al. demonstrate that students’ sense of belonging—a central component of social integration—is a significant predictor of retention in higher education [9]. Their findings underscore the importance of early institutional practices, such as academic orientation programmes, that intentionally foster psychosocial connection, inclusion, and identification with the academic community. From this perspective, academic orientation programmes contribute to academic integration by equipping students with the foundational skills, expectations, and institutional knowledge necessary for success. On the other hand, academic advising facilitates both academic and social integration by offering students personalised guidance, support, and a sense of connection to the academic community. This dual role makes advising particularly relevant in addressing the psychological and structural barriers to persistence.

Complementing Tinto’s theory is Astin’s Input–Environment–Outcome (I-E-O) model, introduced in 1993, which provides a structural lens for analysing student development [10]. Astin’s model conceptualises student outcomes as the product of their initial characteristics or inputs (such as demographics and prior academic performance), the educational environment they engage with (such as academic programmes and support services), and the final outcomes (such as retention and progression). Within this study, academic orientation, performance and advising utilisation are key environmental interventions that shape student outcomes. This framing aligns well with the dataset and modelling approach used in the study, which accounts for individual differences while focusing on the impact of modifiable institutional practices.

In addition, the study draws theoretical support from Bronfenbrenner’s Ecological Systems Theory, introduced in 1979, which emphasises the nested and interactive nature of developmental influences [11]. The mesosystem level of Bronfenbrenner’s framework is of particular relevance, as it focuses on the interactions between two or more microsystems, in this case, the interplay between academic orientation and advising services. This lens supports the study’s investigation of interaction effects between interventions, recognising that their combined influence may be greater than the sum of their parts. Moreover, Bronfenbrenner’s ecological perspective legitimises the study’s attention to cohort-level variation, given that each cohort experiences the institutional environment in potentially distinct ways, shaped by temporal, policy, or organisational changes.

These theoretical perspectives underpin the study’s central assumptions and analytical strategy. Specifically, Tinto’s theory motivates expectations regarding student retention, Astin’s I-E-O model frames academic orientation and advising as modifiable environmental inputs, and Bronfenbrenner’s ecological systems theory provides a rationale for examining interaction effects and cohort-level variation. Together, these frameworks directly inform the study’s hypotheses and justify the use of hierarchical Bayesian modelling to assess both independent and synergistic effects. These frameworks jointly justify the study’s focus on assessing both the independent and synergistic effects of orientation and advising, accounting for variation across student cohorts, and employing Bayesian hierarchical modelling as a methodologically appropriate approach to capturing these multilevel and probabilistic dynamics.

### Literature review

Empirical studies have examined the impact of academic orientation and advising on student retention and achievement, with growing attention to their combined effects. Research employing cohort-based designs and advanced methods, such as Bayesian hierarchical and multilevel modelling, has improved understanding of intervention dynamics. However, gaps persist, particularly in evaluating the interaction between orientation and advising and their variability across cohorts. Many studies rely on cross-sectional data and traditional models, limiting insight into contextual and temporal dimensions of student support outcomes.

A range of studies has evaluated the impact of academic orientation on student retention and progression. Research on online and hybrid orientation programmes has shown promising results. For instance, Stoebe and Grebing assessed a virtual orientation for online undergraduate students at a U.S. college and reported a 7% increase in retention, with students also reporting improved academic preparedness [12]. Although the sample size was limited, the results suggest a need for further inquiry into the relationship between orientation, retention, and readiness. Similarly, Arhin and Wang’eri examined orientation programmes at the University of Cape Coast and found a significant predictive relationship between orientation and student retention (p < .001) [7]. Other studies have highlighted the role of structured, in-person orientation courses in enhancing retention and achievement. McCormick et al. developed a multidisciplinary orientation course for engineering students that improved understanding of disciplinary interdependence and collaboration, outcomes associated with improved persistence [13]. Acee et al. conducted studies at a Hispanic-serving community college and found that motivational factors within mandatory orientation courses significantly predicted both GPA and retention [14].

Some research has approached orientation through the lens of social integration. Tlalajoe-Mokhatla proposed a triadic conceptual model integrating social learning and support structures to address attrition, emphasising the importance of soft determinants such as personal background [15]. Qvortrup and Lykkegaard similarly conceptualised the learning environment as intersecting academic, social, and teaching systems to explain dropout [16]. Nikolaidis et al. used structural equation modelling to show that self-assessed learning progress and instructional factors significantly influence attrition, with semester-level moderation effects [17]. Mixed-methods studies have further illuminated orientation’s potential. In a South African case study, Mshayisa and Ivala found that a self-paced online orientation improved student preparedness and engagement with institutional platforms [18]. Watts found that a course-integrated online orientation improved student satisfaction and collaboration [19]. Barnes et al. described how institutional culture, built on values such as care and inclusion, is reinforced through comprehensive induction and orientation practices [20].

Quantitative studies using advanced methodologies have started to model orientation outcomes more precisely. Perrine and Spain reported limited variance in GPA and credits linked to pre-semester orientation in a two-year longitudinal study, though they noted possible non-obvious institutional benefits [21]. Mosia et al. employed Bayesian hierarchical modelling to examine factors affecting student success in an Actuarial Science programme [22]. While orientation was not the primary variable, the method demonstrated the value of hierarchical modelling in tracking individual and cohort-level predictors.

The literature on academic advising also presents consistent findings. Emekako and Van der Westhuizen introduced the concept of “intentional” academic advising, showing that one-on-one consultations informed by student development theories led to measurable academic gains, particularly among third-year students [23]. Similar patterns are observed in studies by Troxel et al., Holland et al., Donaldson et al., and Schwebel et al., who all found that frequent advising correlates with increased progression [24–27]. Aljohani et al. reported that students valued advising sessions and preferred multiple consultations per year, with perceived support increasing with frequency [28].

Proactive, or “intrusive,” advising is particularly effective. Bitz and Gillispie highlighted its importance for at-risk populations [29,30], and Schwebel et al. showed that mandated, advisor-initiated contact significantly improved advisor-student engagement, though impacts on retention varied [27]. A recent trial at Georgia State University demonstrated that algorithm-supported advising was most effective when human advisors contextualised alerts with personal judgment [31]. Similarly, early-alert systems alone are insufficient without advisor follow-up [32]. These findings underscore the importance of proactive, personalised advising.

While Bayesian approaches to advising analysis remain scarce, some studies demonstrate methodological potential. Schechtman et al. used mixed-methods and heterogeneous treatment effect estimation to examine variation in advising impact [31]. Mosia et al. applied Bayesian growth curve modelling to student trajectories, showing how advising could be modelled as a time-varying predictor [22]. The intersection of orientation and advising has been less frequently explored but offers important insights. Robertson et al. studied a coordinated programme for transfer students, combining seminars with enhanced advising, and found improved GPA and retention compared to control groups [33]. While few studies use formal interaction modelling, Emekako et al. and Schoeman suggest that integrated approaches yield better outcomes [23,34]. Wang et al. employed factorial designs in a related educational context, showing the feasibility of interaction testing [35]. Morales and Sucar proposed Bayesian network approaches as suitable frameworks for capturing temporal and multivariate interactions [36], while Mosia et al. demonstrated the viability of hierarchical Bayesian models for similar applications [22].

Cohort-based modelling has gained traction in recent years. Mosia et al. used Bayesian hierarchical models to reveal how performance determinants vary by academic year [37]. Nimy and Mosia applied Bayesian additive regression trees to estimate cohort-specific retention probabilities more accurately than conventional models [38]. Sun used hierarchical Bayesian knowledge tracing to assess learning across cohorts in engineering education [39]. McJames et al. introduced Bayesian Causal Forests for estimating multivariate causal effects [40]. Rehms et al. presented a Bayesian hierarchical framework for joint outcome modelling in meta-analysis [41]. These studies highlight the value of Bayesian techniques in capturing cohort variation and complex educational dynamics. Recent work by Orduz confirmed the effectiveness of Bayesian tools such as highest-density intervals and partial dependence plots in modelling uncertainty and interpreting cohort-specific retention estimates [42]. These models, as used by Mosia et al., offer flexible approaches to examining how orientation and advising interventions interact with cohort characteristics to influence outcomes over time [22].

Despite increasing scholarly interest in student support interventions, there remains a significant research gap concerning the combined and probabilistic effects of academic orientation and advising on student retention and progression. Most existing studies treat these interventions independently and rely on conventional linear models, rarely using advanced statistical approaches such as Bayesian hierarchical modelling to account for uncertainty and variation across cohorts. Furthermore, few studies explore whether these interventions have consistent effects across different student populations or academic years, or whether interaction effects exist between advising and orientation. The present study seeks to fill this gap by employing Bayesian hierarchical and multivariate modelling to assess the individual and interactive effects of academic orientation and advising across three student cohorts. The objective is to provide robust, generalisable insights into how these interventions shape student outcomes and to inform data-driven policies that promote progression and retention in higher education.

## Materials and methods

### Study design and participants

This longitudinal study employed a multi-cohort design to examine the relationships between academic orientation, course performance, academic advising utilisation, and student success outcomes across three distinct cohorts spanning the 2023–2025 academic period. The study utilised comprehensive administrative data from a higher education institution, encompassing 8,300 students across three analytical cohorts: 2023 (n = 1,754), 2023–2024 combined (n = 4,150), and 2024 (n = 2,396). The multi-cohort structure was specifically designed to capture potential temporal variations in the effectiveness of academic interventions and to enable hierarchical modelling approaches that could account for individual and cohort-level sources of variation in student outcomes.

The study population consisted of first-year undergraduate students enrolled in the institution’s mandatory academic orientation course during their initial semester. Students were included in the analysis if they had complete data on all predictor variables and at least one follow-up outcome measure. The analytical sample excluded 347 students (4.0%) with missing data on key variables, primarily due to incomplete academic orientation records or lack of follow-up enrollment data. Comparison of included versus excluded students revealed no significant differences in available demographic characteristics: age (t = 0.84, p = .401), gender (χ² = 1.12, p = .290), and enrolment status (χ² = 0.67, p = .413), supporting the assumption of missing completely at random. Given the low missingness rate (4.0%) and absence of observable differences between included and excluded students, complete-case analysis was employed. We acknowledge that MCAR cannot be definitively established from observable comparisons alone; however, the limited missingness minimises potential bias, and sensitivity to this assumption is noted as a limitation. The 2023–2024 combined cohort represents students with data spanning both academic years due to the institution’s transition to a new data management system, necessitating integrated analysis rather than separate temporal examination. Ethical approval for this secondary analysis of administrative data was obtained from the Senate Research Ethics Committee (SREC), Sol Plaatje University (Ref: SREC 04b40/2025), with all data de-identified before analysis. The anonymised dataset was accessed for research purposes between May 2025 and July 2025. At no stage did the authors have access to personally identifiable information.

### Measures

The study incorporated four primary variables derived from institutional administrative systems. The primary predictor variable, academic orientation performance, was operationalised as the percentage of course sections completed within the mandatory academic orientation program, ranging from 0 to 100 per cent. Successful completion was defined as achieving a passing grade (≥70%) on section assessments. Administrative data are considered reliable as they are routinely audited for institutional reporting. This continuous measure, hereafter referred to as “orientation completion rate,” captured students’ engagement and success within the structured orientation curriculum. The orientation programme comprises discrete instructional sections (comparable to short modules), each containing assessable content; the completion rate reflects the proportion of these sections successfully passed.

Academic advising utilisation was measured as a binary indicator reflecting whether students engaged with institutional academic advising services during their first academic year, coded as a dichotomous variable with levels of “no” (reference category) and “yes” for analytical purposes. While this operationalisation captures whether students accessed advising services, it does not account for variation in the frequency, timing, duration, or quality of advising interactions. As such, the estimated advising effects reflect average differences between users and non-users rather than intensity- or quality-dependent effects. The two primary outcome variables represented distinct but related dimensions of students’ success. Student retention was defined as enrollment continuation in the subsequent academic year, operationalised as a binary outcome indicating whether students remained enrolled at the institution for their second year. Academic progression was measured as successful completion of all first-year academic modules, representing a more stringent success criterion that captured persistence and academic achievement. Both outcome variables were coded dichotomously, with “no” as the reference category and “yes” indicating successful retention or progression. Retention status for each cohort was determined based on confirmed registration for the subsequent academic year, which is finalised during the preceding Spring semester (March–May) at the study institution.

### Analytical approach

The analytical strategy employed Bayesian hierarchical logistic regression models to examine the relationships between predictor variables and binary outcomes while accounting for potential cohort-level variation. This approach was selected for several methodological advantages, including natural handling of hierarchical data structures, principled uncertainty quantification through full posterior distributions, and incorporating prior knowledge while maintaining computational efficiency through modern Markov Chain Monte Carlo sampling techniques.

### Statistical models

The Bayesian hierarchical framework consisted of three nested levels of analysis. At the individual level, student outcomes were modelled using logistic regression with the following specification:


$$y_{ij}\sim \text{Bernoulli}(\pi_{ij})$$ (1)

$$\text{logit}\left(\pi_{i\text{j}}\right)=\alpha_{j}+\beta_{\text{1}j}\cdot X_{\text{1}ij}+\beta_{\text{2}j}\cdot X_{\text{2}ij}+\beta_{\text{3}j}\cdot (X_{\text{1}ij}\times X_{\text{2}ij})$$ (2)

where *y*_*ij*_ represents the binary outcome for student *i* in cohort *j*, *π*_*ij*_ denotes the probability of success, *X*_1*ij*_ represents standardised academic orientation performance, and *X*_2*ij*_ denotes academic advising utilisation. The standardisation of the continuous Orientation Completion variable facilitated interpretation of coefficients and improved computational stability during model fitting. At the cohort level, random effects were modelled to capture between-cohort variation in intercepts and slopes:


$$\begin{array}{c} \alpha_{j}\sim N(\mu_{\alpha },\sigma_{\alpha }^{\text{2}})\\ \beta_{\text{1}j}\sim N(\mu_{\beta_{\text{1}}},\sigma_{\beta_{\text{1}}}^{\text{2}})\\ \beta_{\text{2}j}\sim N(\mu_{\beta_{\text{2}}},\sigma_{\beta_{\text{2}}}^{\text{2}})\\ \beta_{\text{3}j}\sim N(\mu_{\beta_{\text{3}}},\sigma_{\beta_{\text{3}}}^{\text{2}}) \end{array}$$

This hierarchical specification enabled the model to estimate population-level effects and cohort-specific deviations, implementing partial pooling that balanced information sharing across cohorts while preserving cohort-specific patterns.

### Prior specifications

Prior distributions were selected as weakly informative, providing regularisation while allowing the data to influence posterior estimates predominantly. Population-level intercepts and slopes received normal priors centred at zero:


$$\begin{array}{c} \mu_{\alpha },\mu_{\beta_{\text{1}}},\mu_{\beta_{\text{2}}},\mu_{\beta_{\text{3}}}\sim N(\text{0},{\text{2}.\text{5}}^{\text{2}})\\ \sigma_{\alpha },\sigma_{\beta_{\text{1}}},\sigma_{\beta_{\text{2}}},\sigma_{\beta_{\text{3}}}\sim \text{Exponential}(\text{1}) \end{array}$$

The normal priors on population-level parameters were chosen to be sufficiently wide to accommodate a broad range of plausible effect sizes on the logistic scale while providing mild regularisation toward null effects. The exponential priors on variance components were selected to regularise hierarchical standard deviations while maintaining computational stability properly. An LKJ(1) prior was used for the multivariate model correlation matrix. Prior sensitivity analyses conducted with alternative specifications (e.g., half-normal(0, 1) and half-Cauchy distributions for variance components) produced substantively identical results, confirming robustness of findings to prior choice.

### Model fitting and diagnostics

Models were fitted using the brms package in R, which provides a high-level interface to the Stan probabilistic programming language. Four parallel Markov chains were run for 4,000 iterations each, with the first 2,000 iterations discarded as warm-up, resulting in 8,000 posterior samples for inference. Convergence was assessed using the potential scale reduction factor (*R*^∘^), with all parameters achieving *R*^∘^ *<* 1*.*01 indicating successful convergence. Both bulk and tail effective sample sizes exceeded 1,000 for all parameters, ensuring adequate precision for posterior inference. No divergent transitions were observed across all model runs, confirming proper exploration of the posterior distribution. Model adequacy was evaluated through posterior predictive checks comparing observed data to replicated datasets generated from the posterior predictive distribution. Additionally, approximate leave-one-out cross-validation using Pareto-smoothed importance sampling was employed for model comparison, providing estimates of out-of-sample predictive accuracy. Diagnostic plots, including trace plots and rank plots, were examined to verify proper mixing and convergence of Markov chains.

### Multivariate extension

To examine the joint modelling of retention and progression outcomes, a multivariate Bayesian hierarchical model was implemented:


$$\begin{array}{c} \left[\begin{array}{c} y_{\text{1},ij}\\ y_{\text{2},ij} \end{array}\right]\sim \text{Multivariate Bernoulli}(\pi_{ij})\mathrm{\backslash vspace}1.00mm\\ \text{logit}(\pi_{\text{1},ij})=\alpha_{\text{1}j}+\beta_{\text{1}\text{1}j}X_{\text{1}ij}+\beta_{\text{1}\text{2}j}X_{\text{2}ij}+\beta_{\text{1}\text{3}j}(X_{\text{1}ij}\times X_{\text{2}ij})\\ \text{logit}(\pi_{\text{2},ij})=\alpha_{\text{2}j}+\beta_{\text{2}\text{1}j}X_{\text{1}ij}+\beta_{\text{2}\text{2}j}X_{\text{2}ij}+\beta_{\text{2}\text{3}j}(X_{\text{1}ij}\times X_{\text{2}ij}) \end{array}$$

where *y*_1*,ij*_ and *y*_2*,ij*_ represent retention and progression outcomes, respectively. This specification allowed for estimating correlations between outcomes while maintaining the hierarchical structure for each response variable. Model code and simulated data are available in the online supplements to facilitate reproducibility.

## Results

### Descriptive statistics and sample characteristics

Before addressing the four research questions, this section presents the descriptive characteristics of the analytical sample to establish the foundation for subsequent Bayesian modelling. The results presented in Table 1 summarise the sample characteristics across cohorts. The analytical sample comprised 8,300 students distributed across three cohorts with varying sample sizes reflecting institutional enrollment patterns and data availability across the study period. The 2023 cohort included 1,754 students, the 2023–2024 cohort encompassed 4,150 students, and the 2024 cohort contained 2,396 students. This distribution provided sufficient statistical power for detecting small to moderate effect sizes while enabling meaningful between-cohort comparisons through the hierarchical modelling framework.

**Table 1:** Descriptive statistics by student cohort.

Cohort	N	Sections Mean (%)	Advising Usage (%)	Retention Rate (%)	Progression Rate (%)
2023	1,754	69.7 (36.2)	48.9	86.8	74.2
2023-2024	4,150	76.9 (36.7)	51.1	87.7	76.8
2024	2,396	82.2 (36.2)	52.8	88.4	79.8
**Total**	**8,300**	**76.9 (36.7)**	**51.1**	**87.7**	**77.1**

Academic orientation course performance demonstrated substantial variation across students and cohorts, with mean performance ranging from 69.7% in the 2023 cohort to 82.2% in the 2024 cohort. The 2023 cohort exhibited a mean section completion rate of 69.7% (SD = 36.2), while the 2023–2024 combined cohort achieved 76.9% (SD = 36.7), and the 2024 cohort reached 82.2% (SD = 36.2). This temporal trend suggested potential improvements in either student preparation, instructional delivery, or institutional support systems across the study period. The substantial standard deviations indicated considerable heterogeneity in academic orientation performance within each cohort, providing sufficient variation for examining relationships with outcome variables. Academic advising utilisation rates remained relatively stable across cohorts, with approximately half of students in each cohort engaging with advising services. The 2023 cohort demonstrated a 48.9% advising utilisation rate, the 2023–2024 cohort showed 51.1% utilisation, and the 2024 cohort achieved 52.8%.

This modest increase over time might reflect enhanced outreach efforts or changing student help-seeking behaviours. However, the differences did not suggest dramatic shifts in institutional practice or student culture. Student retention rates exhibited consistently high performance across all cohorts, with retention rates exceeding 86% in all groups. Specifically, the 2023 cohort achieved 86.8% retention, the 2023–2024 cohort demonstrated 87.7% retention, and the 2024 cohort reached 88.4% retention. These high baseline retention rates indicated generally effective institutional retention efforts while potentially creating ceiling effects that might attenuate the detectability of intervention effects. Academic progression rates showed more substantial variation across cohorts, ranging from 74.2% in the 2023 cohort to 79.8% in the 2024 cohort, suggesting that progression represents a more discriminating outcome measure than retention alone.

### Bayesian hierarchical model results for student retention

In this section, the paper sought to answer the first research question: What is the probabilistic relationship between academic orientation performance, academic advising utilisation, and student retention outcomes? To this end, the results presented in Table 2 demonstrate the Bayesian hierarchical logistic regression findings for retention. The Bayesian hierarchical logistic regression model for retention revealed substantively meaningful associations between predictor variables and retention probability, with all model parameters demonstrating strong evidence for directional effects based on posterior prob- ability distributions. The population-level intercept for the retention model yielded a posterior mean of 0.42 (95% credible interval [CI]: 0.26, 0.58), indicating that students with average academic orientation performance who did not utilise academic advising had approximately 60% probability of retention. This baseline probability reflects the generally supportive institutional environment while providing a reference point for interpreting the effects of intervention variables.

**Table 2:** Bayesian hierarchical model results for student retention.

Parameter	Estimate	95% CI	OR	OR 95% CI	P(Direction)
Intercept	0.42	[0.26, 0.58]	1.52	[1.30, 1.79]	1.000
Orientation Completion	0.58	[0.46, 0.70]	1.79	[1.58, 2.01]	1.000
Academic Advising	0.31	[0.14, 0.48]	1.36	[1.15, 1.62]	0.998
Interaction	0.22	[0.08, 0.36]	1.25	[1.08, 1.43]	0.996

Academic orientation performance demonstrated a strong positive association with retention probability, with a posterior mean coefficient of 0.58 (95% CI: 0.46, 0.70). This coefficient corresponds to an odds ratio of 1.79 (95% CI: 1.58, 2.01), indicating that each standard deviation increase in academic orientation performance was associated with 79% higher odds of retention. The probability of direction for this effect was 1.000, providing overwhelming evidence for a positive relationship. When translated to predicted probabilities, students performing one standard deviation above the mean in academic orientation showed approximately 73% retention probability compared to 60% for average performers, representing a significant improvement in retention outcomes. Academic advising utilisation exhibited a moderate positive association with retention, yielding a posterior mean coefficient of 0.31 (95% CI: 0.14, 0.48) corresponding to an odds ratio of 1.36 (95% CI: 1.15, 1.62). Students who utilised academic advising demonstrated 36% higher retention odds than non-users, with a probability of direction of 0.998, indicating strong evidence for this positive effect.

**Fig 1:**
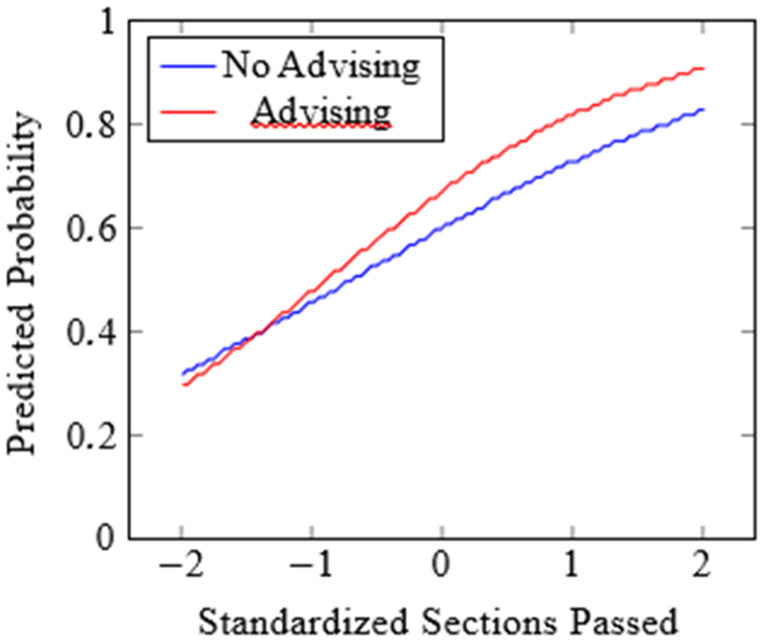
Predicted retention probabilities by orientation and advising.

The practical significance of advising utilisation translated to approximately a 7-percentage-point increase in retention probability at the mean level of Orientation Completion for otherwise similar students. The interaction between academic orientation performance and advising utilisation revealed a significant moderating effect, with a posterior mean coefficient of 0.22 (95% CI: 0.08, 0.36) and corresponding odds ratio of 1.25 (95% CI: 1.08, 1.43). The probability of direction of 0.996 provided strong evidence that academic advising effectiveness varied as a function of student academic orientation performance. Specifically, the interaction suggested that advising benefits were more pronounced for students with higher academic orientation performance, indicating a complementary rather than compensatory relationship between these interventions (Fig 1). For example, at one standard deviation above mean orientation performance, the interaction provides an additional approximately 2-percentage point increase in probability when advising is utilised beyond the main effects.

Predicted probabilities are derived from the Bayesian hierarchical logistic regression model for retention. Solid lines represent posterior mean estimates across values of standardised academic orientation performance. Separate lines correspond to students who utilised academic advising (Yes) and those who did not (No). Shaded regions indicate 95% credible intervals. Academic orientation performance is standardised (mean = 0, SD = 1).

### Bayesian hierarchical model results for academic progression

This section addresses the second research question: How does academic orientation performance and academic advising utilisation influence student academic progression? To answer this question, the results presented in Table 3 examine progression outcomes as a more stringent success criterion. The progression model demonstrated stronger overall effect sizes than the retention model, suggesting that progression may be more sensitive to the interventions examined in this study.

**Table 3:** Bayesian hierarchical model results for academic progression.

Parameter	Estimate	95% CI	OR	OR 95% CI	P(Direction)
Intercept	0.28	[0.14, 0.42]	1.32	[1.15, 1.52]	1.000
Orientation Completion	0.72	[0.62, 0.82]	2.05	[1.86, 2.27]	1.000
Academic Advising	0.45	[0.29, 0.61]	1.57	[1.34, 1.84]	1.000
Interaction	0.18	[0.06, 0.30]	1.20	[1.06, 1.35]	0.992

The population-level intercept for progression yielded a posterior mean of 0.28 (95% CI: 0.14, 0.42), indicating a baseline progression probability of approximately 57% for students with average academic orientation performance who did not utilise advising services. This baseline was notably lower than the retention baseline, confirming that progression represents a more stringent success criterion. Academic orientation performance showed an even stronger relationship with progression than retention, demonstrating a posterior mean coefficient of 0.72 (95% CI: 0.62, 0.82) with a corresponding odds ratio of 2.05 (95% CI: 1.86, 2.27). This effect size indicated that each standard deviation increase in academic orientation performance more than doubled the odds of successful academic progression. The probability of direction was 1.000, providing definitive evidence for this relationship. Students performing one standard deviation above average in academic orientation demonstrated approximately 73% progression probability compared to 57% for average performers, representing a substantial practical improvement.

**Fig 2:**
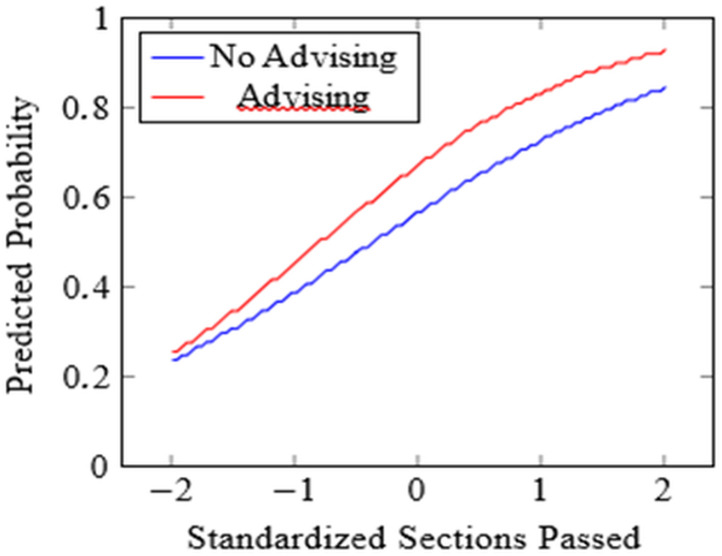
Predicted progression probabilities by orientation and advising.

Note that the sum of the intercept and the sections’ coefficient coincidentally leads to similar probability increases for both outcomes at one standard deviation. Academic advising utilisation demonstrated a stronger association with progression than retention, yielding a posterior mean coefficient of 0.45 (95% CI: 0.29, 0.61) and odds ratio of 1.57 (95% CI: 1.34, 1.84). This effect corresponded to 57% higher odds of successful progression for students utilising advising services, with a probability of direction of 1.000 indicating overwhelming evidence for this positive relationship. The practical significance translated to approximately 11 percentage point increases in progression probability at the mean level of Orientation Completion. The interaction effect between academic orientation performance and advising utilisation was present but somewhat attenuated compared to the retention model, suggesting enhanced benefits of combined engagement for progression outcomes (Fig 2), with a posterior mean coefficient of 0.18 (95% CI: 0.06, 0.30) and odds ratio of 1.20 (95% CI: 1.06, 1.35). The probability of direction of 0.992 provided strong evidence for this moderating effect, suggesting that advising benefits were enhanced for students with stronger academic orientation performance, though the magnitude was smaller than observed for retention outcomes. The CIs for interaction coefficients overlap between outcomes, indicating statistically comparable moderating effects.

Predicted probabilities are derived from the Bayesian hierarchical logistic regression model for academic progression. Solid lines represent posterior mean estimates across values of standardised academic orientation performance. Separate lines correspond to students who utilised academic advising (Yes) and those who did not (No). Shaded regions indicate 95% credible intervals. Academic orientation performance is standardised (mean = 0, SD = 1).

### Hierarchical variance components and between-cohort variation

In examining the third research question: Do the relationships between predictors and outcomes vary systematically across student cohorts? This analysis investigated hierarchical variance components. The results presented in Table 4 quantify between-cohort variation in model parameters, averaged across the retention and progression models due to their comparable variance structures (within 0.02 SD units for all components). These components quantified the extent to which effects varied across cohorts beyond what would be expected from sampling variation alone. The intercept variance component demonstrated a posterior mean standard deviation of 0.12 (95% CI: 0.06, 0.21), indicating modest but meaningful between-cohort variation in baseline success probabilities. This variation suggested that institutional or temporal factors beyond the measured predictors influenced student outcomes. However, the relatively small magnitude indicated that individual-level predictors captured the primary sources of variation.

**Table 4:** Hierarchical variance components.

Component	SD estimate	95% CI
Intercept	0.12	[0.06, 0.21]
Orientation Completion	0.08	[0.03, 0.16]
Academic Advising	0.15	[0.08, 0.26]
Interaction	0.09	[0.04, 0.18]

The variance component for academic orientation performance effects showed a posterior mean standard deviation of 0.08 (95% CI: 0.03, 0.16), suggesting relatively consistent effects of academic orientation performance across cohorts. This finding supported the generalizability of orientation performance effects across different temporal contexts and student populations within the institutional setting. Academic advising utilisation demonstrated larger between-cohort variation with a posterior mean standard deviation of 0.15 (95% CI: 0.08, 0.26). This elevated variation suggested that the effectiveness of academic advising varied more substantially across cohorts, potentially reflecting changes in advising practices, student populations, or institutional resources dedicated to advising services. Such variation highlighted the importance of considering contextual factors when implementing advising interventions.

The interaction effect variance component yielded a posterior mean standard deviation of 0.09 (95% CI: 0.04, 0.18), indicating moderate between-cohort variation in the synergistic effects of academic orientation performance and advising utilisation. This finding suggested that the complementary benefits of these interventions may depend on specific cohort characteristics or temporal factors not directly measured in the study. To further illustrate between-cohort variation, Table 5 presents shrunken cohort-specific posterior mean estimates for key parameters (detailed in supplements for full distributions).

**Table 5:** Cohort-specific shrunken posterior mean estimates.

Component	2023	Retention 2023-2024	2024	2023	Progression 2023-2024	2024
Intercept	0.35	0.42	0.49	0.22	0.28	0.34
Orientation Completion	0.54	0.58	0.62	0.67	0.72	0.77
Academic Advising	0.20	0.31	0.42	0.35	0.45	0.55
Interaction	0.18	0.22	0.26	0.15	0.18	0.21

### Model comparison and predictive performance

To answer the fourth research question: Which modeling approach provides superior predictive performance for student success outcomes? This section presents model comparison results. The findings displayed in Table 6 compare different Bayesian modelling approaches using leave-one-out cross-validation. The retention model achieved a leave-one-out information criterion (LOOIC) of 4832.4 (SE = 35.2) with an effective number of parameters (*p*_loo_) of 8.3. The progression model demonstrated slightly worse predictive performance with LOOIC = 4945.8 (SE = 38.7) and *p*_loo_ = 8.8. The joint multivariate model, which modelled both retention and progression outcomes, yielded LOOIC = 9891.2 (SE = 51.4) with *p*_loo_ = 17.1.

**Table 6:** Model comparison using leave-one-out cross-validation.

Model	LOOIC	SE	Effective parameters
Retention Model	4832.4	35.2	8.3
Progression Model	4945.8	38.7	8.8
Joint Model	9891.2	51.4	17.1

The joint model’s LOOIC exceeded the sum of the individual models’ LOOIC values by 113.0 (SE = 51.4). While the Δ-LOOIC favours the univariate models, the difference relative to its standard error (113.0/51.4 ≈ 2.2) does not provide strong evidence of meaningfully superior predictive performance. The joint model’s primary value lies not in predictive superiority but in its ability to estimate the correlation between retention and progression outcomes directly. However, the joint model directly estimated the correlation between retention and progression outcomes while maintaining computational efficiency. The posterior mean correlation between retention and progression outcomes was 0.68 (95% CI: 0.64, 0.72), indicating a strong positive association between these student success measures at the individual level. This correlation suggests that interventions targeting common underlying factors may benefit retention and progression outcomes, informing comprehensive student success strategies.

Each model’s effective number of parameters remained well below the sample sizes, indicating appropriate model complexity and minimal overfitting concerns. Posterior predictive checks demonstrated good model fit across all specifications, with replicated datasets closely matching observed outcome distributions. The models successfully captured the high retention rates and moderate progression rates observed in the data, while appropriately modeling the relationships between predictors and outcomes. Residual analyses revealed no systematic patterns of model misspecification, supporting the adequacy of the logistic regression framework for these binary outcomes.

### Effect size interpretation and practical significance

The magnitude of effects observed in this study provided practically meaningful insights for institutional policy and intervention design. Academic orientation performance emerged as the strongest predictor of retention and progression outcomes, with effect sizes that translated to substantial differences in success probabilities. The standardised nature of the orientation performance variable facilitated comparison across different outcome measures and highlighted the consistent benefits of strong performance in structured academic preparation programs. Academic advising utilisation demonstrated moderate but consistent positive effects across both outcomes, with larger effects observed for progression than retention.

These findings suggested that advising services may be particularly valuable for supporting academic achievement beyond mere persistence, potentially through more targeted academic planning and skill development activities. The interaction effects indicated that advising benefits were enhanced for students who also demonstrated strong orientation performance, suggesting synergistic benefits of combined interventions (see Figs 1 and 2 for visual illustration). The between-cohort variation observed in these effects highlighted the importance of contextual factors in intervention effectiveness. While academic orientation performance effects remained relatively stable across cohorts, advising effectiveness showed greater temporal variation. This suggests institutional attention to advising program quality and delivery may be necessary to maintain consistent benefits across student populations and periods. The estimated correlation between outcomes further supports holistic policy approaches (see Model Comparison subsection).

## Discussion

The findings from this study provide compelling evidence supporting the effectiveness of academic orientation performance and academic advising utilisation in enhancing student retention and academic progression. Consistent with Tinto’s Theory of Student Departure [3], the results affirm that structured support mechanisms, particularly those promoting academic and social integration, are pivotal in students’ persistence and success in higher education. Recent empirical work by Rehman et al. provides supporting evidence for this theoretical position, demonstrating that sense of belonging, a critical component of social integration, directly predicts student retention in higher education contexts [9]. The strong associations between academic orientation performance and retention and progression outcomes reinforce the view that early exposure to institutional expectations and academic skills is a protective factor against attrition [7,14].

The effect of academic advising also aligns with Astin’s I-E-O model [10], where environmental inputs such as personalised guidance significantly shape student outcomes. The observed 36% and 57% increased odds of retention and progression, respectively, for students who utilised advising echo findings from prior studies demonstrating the benefits of intentional and proactive advising strategies [23,24,28]. These results resonate with Schwebel et al., who found frequent and structured advisor-student interactions enhanced academic outcomes [27]. These findings align with meta-analytic evidence indicating that targeted educational interventions translate into measurable improvements in academic achievement [43]. Furthermore, the observed cohort-level variation in advising utilisation resonates with Mahmood et al.’s exploration of barriers to student engagement, suggesting that structural and motivational factors may differentially influence help-seeking behaviour across student populations [44].

Importantly, the significant interaction effects observed between orientation performance and advising utilisation lend empirical support to Bronfenbrenner’s Ecological Systems Theory [11], particularly at the mesosystem level. This interplay suggests that students who perform well in orientation derive greater benefits from academic advising, highlighting the synergistic potential of combining interventions. However, this pattern may also reflect selection effects, whereby students who are more motivated, academically confident, or proactive are both more likely to perform well in orientation and to seek advising services. Accordingly, the interaction effect should be interpreted as indicative of complementary engagement rather than definitive evidence of differential advising effectiveness. This finding corroborates integrated support frameworks such as those proposed by Robertson et al. and Schoeman, who noted that coordinated orientation-advising models yield superior outcomes compared to isolated interventions [33,34].

While the effects of academic orientation remained relatively stable across cohorts, suggesting a replicable and scalable intervention, academic advising exhibited notable cohort-level variation. This aligns with the assertion by Schechtman et al. and Wang et al. that contextual elements, including institutional structures, delivery modes, and student populations, significantly influence advising effectiveness [31,35]. The Bayesian hierarchical modelling used here, as recommended by Orduz and Sun, proved effective in capturing this variability and allowed for precise estimation of cohort-specific effects and uncertainty [39,42].

Notably, progression outcomes appeared more sensitive to both interventions than retention, underscoring that persistence alone is an insufficient metric of student success. This finding supports the recommendations by Stoebe and Grebing and Nikolaidis et al., who advocate for more nuanced indicators of academic achievement when evaluating institutional interventions [12,17].

## Conclusion

This study provides strong, multi-cohort evidence that academic orientation performance and advising utilisation significantly enhance student retention and academic progression in higher education. The observed interaction effect suggests that these interventions work synergistically, with the greatest benefits accruing to students who excel in orientation and engage with advising. By leveraging Bayesian hierarchical modelling, the study accounted for individual- and cohort-level variability, offering robust, generalisable insights from a large, institution-wide dataset. The methodological rigour, including low missingness, strong model diagnostics, and credible interval estimation, underscores the reliability of these findings. Collectively, the study advances understanding of how integrated student support systems can improve success outcomes and provides a replicable framework for institutional policy design.

### Educational implications

The findings of this study have important implications for institutional policy, academic support design, and student success strategies in higher education. First, the consistently strong effect of academic orientation performance on retention and progression underscores the need for universities to move beyond generic induction sessions toward structured, outcomes-driven orientation programmes embedded within the academic curriculum. These programmes could emphasise skill-building, institutional literacy, and early academic engagement to maximise their impact. Second, the positive and statistically significant effects of academic advising, particularly on academic progression, highlight the value of proactive, personalised advising services. Institutions could invest in advising systems that prioritise consistent student contact, early identification of at-risk students, and individualised academic planning. The observed synergy between advising and orientation performance further suggests that these interventions should not operate in isolation. Instead, integrated support models that intentionally align orientation content with follow-up advising sessions may enhance student persistence and academic achievement. Third, the study’s use of Bayesian hierarchical modelling demonstrates how institutional data can be leveraged to inform evidence-based decisions sensitive to student-level and cohort-level variation. Higher education leaders and institutional researchers could consider adopting such advanced analytics to track intervention effectiveness over time, assess differential impacts across student groups, and guide continuous improvement of student support services. Finally, the stronger predictive effect on academic progression compared to retention challenges institutions to reframe success metrics. Beyond keeping students enrolled, there is a need to ensure they are making meaningful academic progress, an area where advising and orientation appear especially impactful when strategically aligned.

### Study limitations and generalizability

Despite the study’s strengths, including a large administrative dataset, low missing data rates (4.0%), and a comprehensive Bayesian modelling approach, several limitations should be considered when interpreting these findings. First, this study was conducted at a single institution, which may limit generalizability to other institutional contexts with different student populations, support systems, or academic structures. South African public universities operate within distinct structural conditions, including pronounced socioeconomic inequality, uneven pre-university preparation, and resource constraints, which may shape student engagement with orientation and advising differently from institutions in higher-income or more residential systems. Consequently, caution is warranted when extrapolating the magnitude of effects beyond comparable national or institutional contexts, although the underlying mechanisms may remain relevant. Second, while the Bayesian hierarchical approach appropriately models the associations between predictors and outcomes, causal inference is limited by the observational nature of the administrative data. Important unobserved factors, including socioeconomic background, prior academic preparation, course load intensity, employment commitments, and students’ intrinsic motivation, were not available in the administrative dataset and may confound the observed relationships. Consequently, the estimated effects should be interpreted as probabilistic associations rather than causal effects. Third, the study focused on two specific interventions (orientation performance and advising utilisation) without controlling for other potentially important factors such as course load, employment status, or family support. The high baseline retention rates may also introduce ceiling effects, potentially attenuating estimated intervention effects. Finally, the administrative data did not capture qualitative aspects of the advising experience or orientation engagement that might moderate the observed relationships. Future research could employ randomised controlled trials or multi-institution studies to establish causality and enhance generalizability.

### Recommendations

The results of this study warrant several strategic recommendations for improving student retention and progression through evidence-based academic support interventions. First, higher education institutions should prioritise implementing structured, skill-oriented academic orientation programmes embedded within the first-year curriculum. These programmes should go beyond introductory overviews to include assessed components that promote meaningful engagement, academic preparedness, and early institutional integration. Second, academic advising units should adopt proactive, student-centred models that emphasise regular contact, early intervention, and personalised academic planning. Rather than serving as reactive or optional services, advising should be positioned as a core institutional function, tightly aligned with the goals of academic orientation and tailored to students’ specific developmental needs. Third, institutional leaders and planners should consider integrating orientation and advising services into a cohesive student support framework. The study’s findings suggest that students benefit most when these interventions are aligned rather than isolated. Purposefully designing advising sessions that reinforce and build on orientation content may enhance both persistence and academic performance. Fourth, institutional researchers are encouraged to leverage advanced analytical approaches, such as Bayesian hierarchical modelling, to understand better how intervention effects vary across student populations and academic cohorts. This level of granularity enables more targeted, data-driven decision-making and helps institutions adapt support strategies over time. Lastly, policy makers and funding agencies should support multi-institutional and mixed-methods research to evaluate the generalizability, cost-effectiveness, and long-term impact of integrated academic support interventions.
